# Delving into pyroptosis in atherosclerosis: role, mechanisms and therapeutic opportunities

**DOI:** 10.3389/fimmu.2026.1883597

**Published:** 2026-08-07

**Authors:** Huifang Hou, Qian Cheng, Guilin Wang, Mengyao Zhao, Song Wang, Yaling Yin, Qiong Li, Sebastien Roger, Lin-Hua Jiang

**Affiliations:** 1 Department of Physiology and Pathophysiology and Sino-UK Joint Laboratory of Brain Function and Injury of Henan Province, Henan Medical University, Xinxiang, China; 2 School of Basic Medical Sciences, North Henan Medical University, Xinxiang, China; 3 Henan Key Laboratory of Neurorestoratology and Protein Modification, Henan Medical University, Xinxiang, China; 4 Henan Key Laboratory of Medical Tissue Regeneration, Henan Medical University, Xinxiang, China; 5 Inserm U1327 ISCHEMIA ‘Membrane Signalling and Inflammation in Reperfusion Injuries’, Université de Tours, Tours, France; 6 School of Biomedical Sciences, Faculty of Biological Sciences, University of Leeds, Leeds, United Kingdom

**Keywords:** atherosclerosis, gasdermin, inflammation, oxidative stress, pyroptosis

## Abstract

Atherosclerosis is a chronic inflammatory condition that represents the primary culprit of multiple cardiovascular diseases. Pyroptosis is a modality of programmed cell death that is elicited by, and can in return amplify, inflammatory reactions, and thus has attracted increasing attention for its proatherogenic role. Studies have gathered a body of evidence that supports pyroptosis as an important mechanism that drives atherosclerosis pathogenesis and progression. In this review, we present a comprehensive overview of the current state, particularly the recent progress, in understanding pyroptosis of endothelial cells, macrophages and vascular smooth muscle cells and their role in atherosclerosis. We summarize the pathways that mediate cell pyroptosis and the molecular mechanisms that are mobilized by a diversity of proatherogenic risk factors and pathological conditions to stimulate such pyroptosis pathways. We also discuss the ongoing interest in targeting the pyroptosis-driving pathways and mechanisms as therapeutic strategies to intervene atherosclerosis and related cardiovascular diseases.

## Introduction

Atherosclerosis is a major chronic inflammatory condition that arises from deficient lipid metabolism, excessive lipid deposition and ensuing maladaptive immune reactions, leading to formation of atherosclerotic plaques on the arterial walls that disrupt arterial functions (1–3). Atherosclerosis is the primary culprit of multiple cardiovascular diseases (CVDs), including myocardial infarction, heart attack, coronary artery disease, carotid artery disease, aortic aneurysm, which represent the key causes of morbidity and mortality worldwide (3–6). Decades of research have recognized the contribution of both genetic and non-genetic factors and their interactions to the complex etiology of atherosclerosis that is still not entirely understood (1–3). Endothelial cells (ECs) form the innermost layer or intima of the arterial wall and, for the subendothelial space being the key site for lipoprotein deposition and plaque formation, loss of ECs is a common and critical factor initiating atherosclerosis. With disease progressing, vascular smooth muscle cells (VSMCs) in the middle layer of the arterial wall undergo phenotypic switching, particularly acquirement of an inflammatory-like function and cell death, to promote foam cell formation and accelerate development and rupture of atherosclerotic plaques (7, 8). Moreover, functional alterations and death of tissue-resident macrophages and peripheral macrophages infiltrated into the atherosclerotic plaques further fuel foam cell formation and vascular inflammation, exacerbating the formation of lipid-rich necrotic cores and increasing the plaque vulnerability (9, 10). Cell death, a major form of cell dysfunction or loss of function, occurs in multiple modalities, with each driven by distinct signaling pathways and/or molecular mechanisms (11, 12). Pyroptosis is a modality of programmed cell death (PCD) that is specifically initiated by inflammatory reactions and, importantly, can in return amplify the inflammatory reactions by releasing proinflammatory intracellular contents (13). Given the intrinsic nature of atherosclerosis being an inflammatory disease, pyroptosis has attracted increasing attention for its proatherogenic role (14).

In this review, we present a comprehensive overview of the current state of understanding the role of pyroptosis in atherosclerosis, particularly the recent progress in elucidating the pathways mediating pyroptosis of ECs, macrophages and VSMCs and the molecular mechanisms that are mobilized by a diversity of proatherogenic risk factors and pathological conditions to stimulate such pyroptosis pathways. We start with a concise introduction of the major pathways that mediate pyroptosis. We discuss the recent findings from examining the expression of key proteins in the pyroptosis pathways in tissues from patients with atherosclerosis and related conditions. Next, we elaborate the findings from studies using rodent models that support the important role of pyroptosis in atherosclerosis and the proatherogenic actions of a diversity of well-documented risk factors and pathological conditions and, furthermore, the findings from *in vitro* or mechanistic studies that reveal the pyroptosis-mediated pathways and the molecular mechanisms driving upregulation and activation of such pathways in atherosclerotic settings. Finally, we evaluate the ongoing efforts of targeting pyroptosis-driving pathways and mechanisms to intervene atherosclerosis and related CVDs.

## The major pathways mediating pyroptosis

Pyroptosis was initially described as apoptosis in macrophages induced by infection with *Shigella flexneri* (15) or *Salmonella typhimurium* (16), which however manifested distinctive morphological changes, including swelling and membrane rupture. As it is known nowadays, pyroptosis is a distinct modality of PCD that is elicited by inflammatory reactions (13, 17, 18). The groundbreaking discovery of gasdermin proteins has transformed our understanding of the molecular mechanisms mediating pyroptosis, as well as release of interleukin (IL)-1β and IL-18 (14, 19, 20). For recognition of gasdermin as the exclusive executioner, pyroptosis has been refined as proinflammatory gasdermin-mediated modality of PCD (21). There are six gasdermin proteins in humans, gasdermin A-E (GSDMA-E) and PJVK/DFNB59, whereas the GSDMB homologue is lacking and multiple GSDMA and GSDMC homologues are expressed in mice (22). They are structurally and functionally similar, consisting of a N-terminal domain that forms pyroptotic pores in the plasma membrane, a C-terminal domain that serves a structural mechanism inhibiting pore formation, and a linker region between them that contains cleavage sites for proteases (13, 23). Caspases, a family of cysteine proteases, are the key gasdermin-cleaving proteases, representing a major mediator of pyroptosis (24). Below, we briefly introduce the pyroptosis pathways mediated by caspases in order to facilitate the discussion of pyroptosis in atherosclerosis. Readers are recommended to consult elegantly curated reviews for the wealth of mechanistic insights into pyroptosis (25–30).

**Figure 1:**
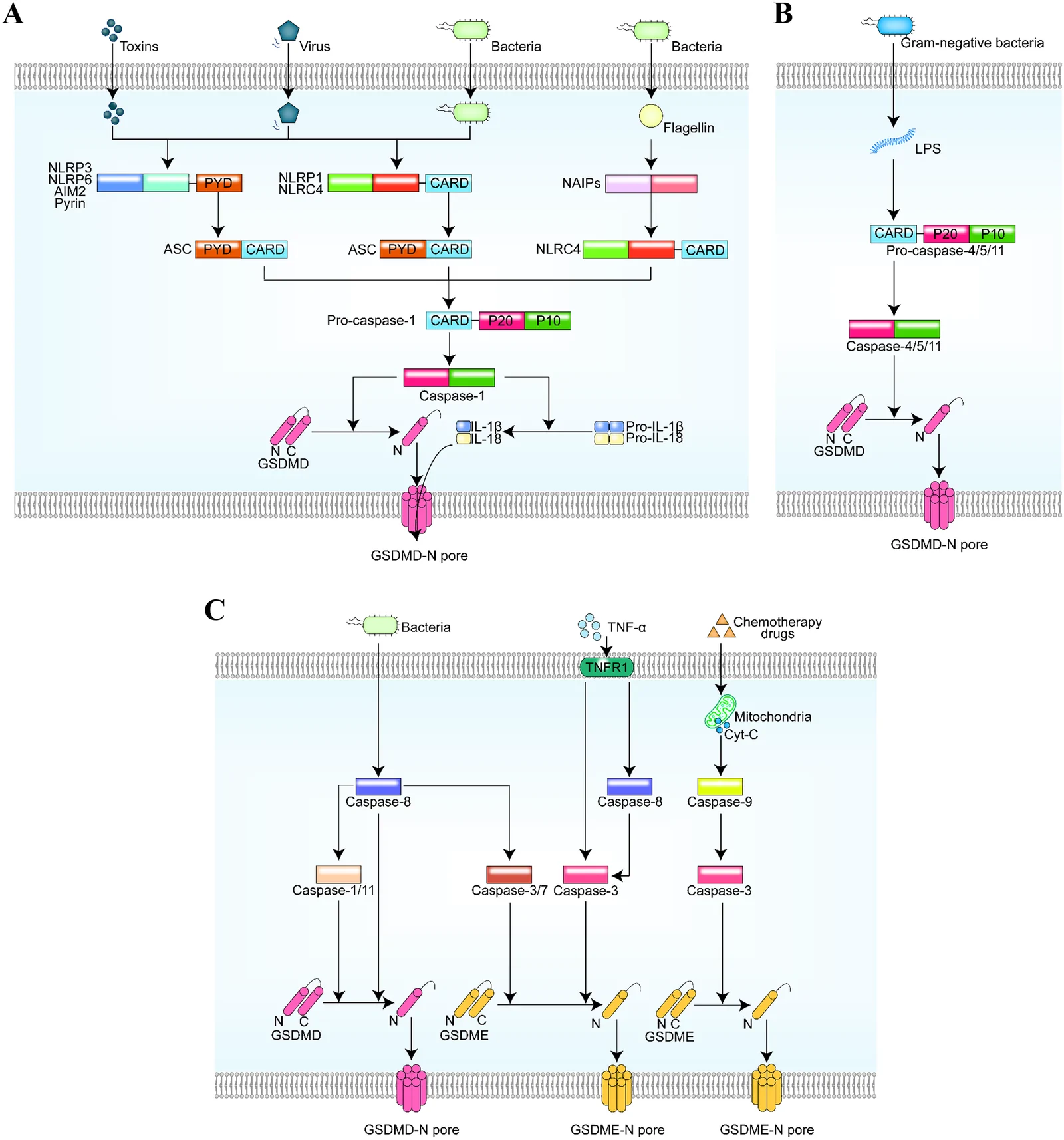
Schematic summary of the major caspase-mediated pyroptosis pathways. **(A)** The canonical pathway. Upon detecting toxins, virus or bacteria, NLRP3, NLRP6, AIM2, Pyrin, NLRP1 and NLRC4 interact with ASC and pro-caspase-1 through the PYD or CARD domain, and NAIPs interacts with NLPC4 and pro-caspase-1, to form and activate the inflammasome that cleaves pro-caspase-1 into p10 and p20 fragments that constitute an active caspase-1. Caspase-1 cleaves GSDMD to generate GSDMD-N, which forms an oligomeric pore to perforate the plasma membrane and execute pyroptosis. Caspase-1 also cleaves pro-IL-1β and pro-IL-18 to generate IL-1β and IL-18, which are released via the GSDMD pore. **(B)** The non-canonical pathway is mediated by human caspase-4/5 or mouse caspase-11. LPS, derived from Gram-negative bacteria interacts with the CARD domain in pro-caspase for autocleavage and activation of caspase. **(C)** The additional pathways engaging other caspases to cleave gasdermin in response to various stimuli. Bacteria (Yersinia or its secreted pathogenic effector YopJ) can activate caspase-8 or further caspase-1 and caspase-11 to cleave GSDMD and, in addition, activate caspase-3/7 to cleave GASDME. TNF-α can via the TNFR1 activate caspase-8 and caspase-3 to cleave GSDME. Chemotherapy drugs can induce mitochondrial ROS generation and Cyt-c release to activates caspase-9 and caspase-3 to cleave GSDME. ASC, apoptosis-associated speck-like protein containing CARD; GSDME or GSDMD, gasdermin E, D; LPS, lipopolysaccharide; IL, interleukin; TNF, tumor necrosis factor; TNFR1, TNF-α receptor 1; Cyt-c, cytochrome c.

There are two well-defined caspase-mediated pyroptosis pathways, which are commonly referred to as the canonical and non-canonical pathways, respectively. Caspase-1 is the signature protease of the canonical pathway (31), and its activation depends upon assembly of a multi-protein complex termed inflammasome, induced upon ligation of a repertoire of pattern recognition receptors (PRRs) by a diversity of molecules with danger-/pathogen-associated molecular patterns (DAMPs/PAMPs) (17). Exemplar PPRs, as illustrated in **Figure 1A**, include NLRP1 [nucleotide-binding and oligomerization domain, leucine-rich repeat (NLR) and pyrin domain containing protein 1], NLRP3, NLRP6, NLRC4 [NLR family caspase activation and recruitment domain (CARD)-containing 4], AIM2 (absent in melanoma 2) and pyrin. Among them, NLRP3, NLRP6, AIM2 and pyrin can form an inflammasome by interacting with the adaptor protein apoptosis-associated speck-like protein containing CARD (ASC) through the pyrin domain (PYD) and, via ASC, further with pro-caspase-1 through the CARD domain (17). Besides the PYD and CARD domains, NLRP1 contains a function-to-find domain (FIIND; not depicted in **Figure 1A**) and, upon self-cleavage of the FIIND domain, can interact with ASC and pro-caspase-1, both mediated by the CARD domain, to assemble an inflammasome. Similarly, NLRC4 can form an inflammasome with ASC and pro-caspase-1 through CARD-mediated interactions. Alternatively, in the absence of ASC, NLRC4 can interact with one of the NLR family apoptosis inhibitory proteins (NAIPs) that function as cytosolic receptors for specific bacterial proteins and then with pro-caspase-1, through the CARD domain, to form an inflammasome. Activation of these inflammasomes promotes proteolytic cleavage of pro-caspase-1 to generate p10 and p20 fragments that constitute the active caspase-1. GSDMD was the firstly identified gasdermin as the substrate of caspase-1 that cleaves human GSDMD at Asp^275^ or mouse GSDMD at the equivalent position Asp^276^ in the linker region (32). The resultant GSDMD-N fragments are oligomerized to form pores to perforate the plasma membrane (33).

The non-canonical pathway utilizes caspase-4 and caspase-5 in humans, or caspase-11, the paralogue of caspase-4 (and there is no counterpart of caspase-5) in mice (**Figure 1B**). As the best elucidated mechanism activating these caspases, lipopolysaccharide (LPS), a component of Gram-negative bacterial wall that can potently induce inflammatory reactions, interacts via its lipid A moiety with the CARD domain of pro-caspase to promote autocleavage for activation (34, 35). Like caspase-1, caspase-4 and caspase-5 cleave human GSDMD at Asp^275^ and capase-11 mouse GSDMD at Asp^276^ to generate the pore-forming GSDMD-N fragment.

Evidence shows other members of the caspase family, including the classical apoptotic caspases, can cleave gasdermin and execute pyroptosis, particularly in response to infection, proinflammatory factors or treatment with anti-tumor drugs (**Figure 1C**). For example, exposure of macrophages to bacterial Yersinia or its secreted pathogenic effector YopJ, when TAK1 is inhibited, can induce pyroptosis by promoting receptor-interacting protein kinase 1 (RIPK1)-dependent activation of caspase-8 to cleave GSDMD (36, 37) and, in addition, induce activation of caspase-1 and caspase-11 to cleave GSDMD or caspase-3 and caspase-7 to cleave GASDME (38). Tumor necrosis factor (TNF)-α, the proinflammatory cytokine that induces sarcopenia among the elderly, via its cognate TNF receptor 1 (TNFR1), can induce sequential activation of caspase-8 and caspase-3 and cleavage of GSDME to execute pyroptosis in skeletal muscle cells (39). Miltirone, a phenanthrene-quinone derivative with an anti-tumor activity, can promote mitochondrial ROS generation and release of cytochrome-c (Cyt-c) that activates caspase-9 and caspase-3 to cleave GSDME and evoke pyroptosis in hepatocellular HepG2 and Hepa1–6 cells (40). Cisplatin, a chemotherapy drug, can activate caspase-3 in a similar mechanism to cleave GSDME and induce pyroptosis in esophageal cancer cells (41).

It is worth mentioning nerve injury-induced ninjurin (NINJ) proteins, particularly NINJ1 for its role in inducing plasma membrane rupture (PMR), downstream of lytic cell death such as pyroptosis (42). During inflammasome-driven pyroptosis, while the pores formed by GSDMD or other pore-forming proteins mediate efflux of small proinflammatory molecules and cytokines, PMR caused by high-order polymerization of NINJ1 permits release of large proinflammatory molecules (29).

## Pyroptosis in atherosclerosis: evidence from examining patient tissues

Several recent studies have examined the level of proteins participating in the pyroptosis pathways in tissues from patients with atherosclerosis or associated conditions, providing evidence to support pyroptosis and its role in atherosclerosis (43–51). Increased NLRP3 expression, caspase-1 activation and GSDMD cleavage were consistently observed in aortic atherosclerotic tissues (46, 48–51). In particular, the pore-forming GSDMD-N was detected in atherosclerotic plaques (44) or, more specifically, in CD31-positive ECs in human coronary arteries from autopsy samples in the advanced atherosclerotic plaques, but not in the early plaques or plaque-free tissues (43), a finding that implicates pyroptosis of ECs in atherosclerosis, especially in the advanced stage. The expression of caspase-3 and GSDME was also upregulated and their interactions were heightened in atherosclerotic plaques in tissues from patients receiving carotid endarterectomy and, moreover, there was an increase in the number of pyroptotic macrophages and also in the level of lipid core formation in atherosclerotic plaques (47). Upregulated expression of NLRP3 and ASC and activation of caspase-1 were also documented in VSMCs in the plaques from symptomatic carotid artery stenosis patients (45). Thus, accumulating evidence indicates that pyroptosis occurs to ECs, macrophages and VSMCs and implicates such cell death in the pathogenesis and progression of atherosclerosis. In patients with coronary artery disease, the NINJ1 expression was upregulated in aortas with atherosclerotic plaques, particularly in CD68-positive macrophages (52). More studies are required to define the contribution of pyroptosis of different cell types in different stages of atherosclerosis and gain insights into the pathways that mediate pyroptosis and the molecular mechanisms that drive activation of such pyroptosis pathways.

## Pyroptosis in atherosclerosis: roles, pathways and mechanisms

Rodent models are useful in interrogating disease mechanisms, and studies using rodent models have demonstrated an important role of pyroptosis in mediating atherosclerosis, particularly in the proatherogenic actions of a diversity of well-documented risk factors and pathological conditions. In addition, studies using cultured cells have shed light on the pyroptosis pathways and the molecular mechanisms that stimulate such pyroptosis pathways.

### Pyroptosis in rodent hyperlipidemia-induced models of atherosclerosis

Hyperlipidemia, arising from deficient lipid metabolism and abnormal lipid deposition, represents the key risk factor of atherosclerosis in humans. Consistently, induction of hyperlipidemia in mice, by genetic deletion of the expression of apolipoprotein E (ApoE) and low-density lipoprotein receptor (LDLR), the main regulators of lipid metabolism, can induce prominent atherosclerosis-related phenotypes (53, 54). Thus, the ApoE^−/−^ mice and the LDLR^−/−^ mice have been used as disease models to examine the role of pyroptosis in atherosclerosis induced by hyperlipidemia on its own or together with other proatherogenic risk factors or pathological conditions (**Table 1**).

**Table 1:** Studies examining pyrotosis and its role in atherosclerosis using mouse models.

Risk factor/condition	Model	Summary of key findings	Ref
Hyperlipidemia	HDF-fed ApoE^–/–^ mice	* The GSDMD expression was upregulated in atherosclerotic aortas and in macrophages; * Deletion of the GSDMD expression reduced atherosclerotic plaque formation.	(48)
		* Infiltration of macrophages into atherosclerotic aortas was increased; * The expression of NLRP3 and GSDMD was upregulated, and GSDMD cleavage was increased in VMSCs in atherosclerotic aorta;	(60)
		* The caspase-1 expression was upregulated, and caspase-1 activation and GSDMD cleavage were increased in atherosclerotic aortic root.	(49)
		* The NLRP3 expression was upregulated, and caspase-1 activation and GSDMD cleavage were increased in ECs in atherosclerotic aortic tissues.	(88)
		* The expression of NLRP3, GSDMD and caspase-1 was upregulated, and GSDMD cleavage was increased in ECs in atherosclerotic aortas.	(59)
		* Caspase-1 activation and GSDMD cleavage were increased in macrophages in atherosclerotic aortic root.	(63)
		* The expression of NLRP3, caspase-1 and GSDMD was upregulated in atherosclerotic aortic root.	(50)
		* The expression of NLRP3 and GSDMD was upregulated in macrophages in atherosclerotic aortas.	(129)
		* Cell death was observed in atherosclerotic plaques; * The expression of GSDME and caspase-3 was upregulated in atherosclerotic aortas; * The GSDME expression was upregulated in macrophages from atherosclerotic tissue; * Deletion of the GSDME expression reduced atherosclerotic plaque formation.	(47)
		* The NLRP3 expression was upregulated, and caspase-1 activation and GSDMD cleavage were increased in fibrous cap and VSMCs in atherosclerotic aortic root.	(55)
		* The expression of NLRP3 and caspase-1 was upregulated in atherosclerotic aortic sinuses; * Infiltration of macrophages into atherosclerotic plaques was elevated; * The caspase-1 expression in macrophages was upregulated in atherosclerotic aortic tissues.	(56)
		* The NLRP3 expression was upregulated, and caspase-1 activation and GSDMD cleavage were increased in atherosclerotic aorta;	(57)
		* The expression of NLRP3, ASC and caspase-1 was upregulated, and GSDMD cleavage was increased in atherosclerotic aorta; * Infiltration of macrophages into atherosclerotic plaques was elevated; * The localization/expression of NLRP3 in macrophages was increased in atherosclerotic aorta.	(58)
		* The expression and cleavage of GSDMD and GSDME were enhanced in atherosclerotic aorta.	(61)
Hyperlipidemia	HFHC diet-fed ApoE^–/–^ mice	* Caspase-11 activation and GSDMD cleavage were increased in atherosclerotic aorta; * Infiltration of macrophages into atherosclerotic aortic arteries was elevated; * The expression of caspase-11 and GSDMD in macrophages was upregulated in atherosclerotic plaque; * Deletion of the caspase-11 expression or knockdown of the GSDMD expression reduced atherosclerotic plaque formation.	(64)
Hyperlipidemia	HFD&HAD-fed ApoE^–/–^ mice	* The expression of NLRP3, caspase-1 and GSDMD was upregulated in atherosclerotic aortic tissues; * EC death was observed in atherosclerotic plaques.	(92)
	WTD-fed ApoE^–/–^ mice	* The expression and cleavage of GSDME were increased in ECs in atherosclerotic aortas; * Deletion of the GSDME expression reduced atherosclerotic plaque formation.	(62)
	WTD-fed LDLR^–/–^ mice	* The activation of caspase-1 and caspase-11, and GSDMD cleavage were increased in atherosclerotic aortas; * Infiltration of macrophages into atherosclerotic aortas was elevated; * Deletion of the GSDMD expression alleviated infiltration of macrophages and plaque formation.	(66)
	WTD-fed JAK2^VF^ LDLR^–/–^ mice	* Depletion of the AIM2 or GSDMD expression reduced formation of necrotic cores and atherosclerotic plaques.	(67)
Hyperuricemia	HFD&HAD-fed ApoE^–/–^ mice	* The expression of NLRP3, caspase-1 and GSDMD was upregulated in atherosclerotic aortic tissues; * EC death was observed in atherosclerotic plaques.	(92)
HHcy	HDHM diet-fed ApoE^–/–^ mice	* The expression of NLRP3, caspase-1 and GSDMD was upregulated in macrophages in atherosclerotic aortic roots and vessels.	(94)
		* The expression of NLRP3, caspase-1 and GSDMD was upregulated, and caspase-1 activation was increased in atherosclerotic aortic root; * The NLRP3 expression was upregulated in macrophage in atherosclerotic plaques.	(95)
Diabetes	HFD-fed ApoE^–/–^ mice with STZ-induced diabetes	* The expression of NLRP3 and caspase-1 was upregulated in atherosclerotic aortic tissues; * Infiltration of macrophage and the expression of inflammatory factors were increased in atherosclerotic aortic tissues.	(101)
		* The expression of NLRP3 and ASC was upregulated, and GSDMD cleavage was increased in atherosclerotic aortic tissues.	(100)
	WTD-fed ApoE^–/–^ mice with STZ-induced diabetes	* The expression of NLRP3, ASC and GSDMD was upregulated in atherosclerotic aortic tissues; * The expression of NLRP3 and ASC was upregulated in ECs in atherosclerotic aortic tissues.	(99)
Metabolites	Neu5Ac-treated ApoE^−/−^ mice	* The NLRP3 expression was upregulated in CD31-positive ECs in aortic roots.	(109)
	TMAO-treated ApoE^−/−^ mice	* The NLRP3 expression was upregulated in ECs of atherosclerotic aortic root.	(108)
LSS	HFD-fed ApoE^−/−^ mice with LSS	* The intimal NLRP3 expression was upregulated in atherosclerotic aortic arch.	(111)
Cigarette smoking	ApoE^−/−^ mice exposed to nicotine	* Cell death was induced in aortic tissue; * The expression of NLRP3 and ASC was upregulated, and caspase-1 activation and GSDMD cleavage were increased in aortic tissue.	(118)
		* Cell death was induced in aortic ECs; * The expression of NLRP3, ASC and caspase-1 was upregulated in aortic ECs.	(117)
		* The expression and activation of caspase-1 were increased in macrophages of atherosclerotic aortas and aortic root.	(116)
Infection	HFD-fed ApoE^−/−^ mice injected with LPS	* The expression of GSDMD, caspase-1 and caspase-11 was upregulated, and GSDMD cleavage was increased in atherosclerotic aortic roots; * The GSDMD expression was upregulated in ECs in atherosclerotic aortic roots; * Endothelial-specific deletion of the GSDMD expression reduced atherosclerotic plaque formation.	(70)
	WT mice infected with mouse CMV	* AIM2 activation and cleavage of GSDMD and GSDME were increased in endothelium and mesothelium.	(124)

HHcy, hyperhomocysteinemia; LSS, low shear stress; HFD, high fat diet; ApoE, apolipoprotein E; HFHC, high fat and high cholesterol; WTD, western type diet; HAD, high adenine diet; LDLR, low-density lipoprotein receptor; STZ, streptozotocin; TMAO, trimethylamine N-oxide; LPS, lipopolysaccharide; CMV, cytomegalovirus; GSDMD/E, gasdermin D or E; EC, endothelial cells; VSMC, vascular smooth muscle cells; NLRP3, nucleotide-binding and oligomerization domain, leucine-rich repeat (NLR) and pyrin domain containing protein 3; ASC, apoptosis-associated speck-like protein containing CARD; AIM2, absent in melanoma 2.

One of the recurrent findings from examining the issues from the ApoE^−/−^ mice fed with high fat diet (HFD) is an increase in the expression and/or activation of pyroptosis-driving proteins, similar to what described above in human atherosclerotic tissues. Thus, the expression of NLPR3 (49, 55–60), ASC (58), caspase-1 (49, 56, 58, 59), caspase-3 (47), GSDMD (48, 49, 51, 55, 57–61) and GSDME (47, 61, 62) in atherosclerotic tissues, particularly, the expression of NLRP3 and GSDMD in macrophages (48, 50, 58, 63) and VSMCs (55, 60), and GSDME in macrophages (47) was upregulated. Furthermore, both caspase-1 activation and cleavage of GSDMD or GSDME were detected in atherosclerotic tissues (55, 57, 58, 61). Importantly, the formation of necrotic cores and atherosclerotic plaques in the HFD-fed ApoE^−/−^ mice was alleviated by deleting the GSDME expression (47, 62). Similarly, upregulated expression of caspase-11 and GSDMD, caspase-11 activation and GSDMD cleavage were observed in the atherosclerotic aorta in the ApoE^–/–^ mice with a high fat and high cholesterol (HFHC) diet (64). In such HFHC diet-fed ApoE^–/–^ mice, atherosclerotic plaque formation and infiltration of macrophages into the plaques were lessened by deleting the expression of caspase-1, caspase-11 or GSDMD (49, 64). These findings compellingly support a vital role of hyperlipidemia-induced GSDMD/GSDME-mediated pyroptosis in atherosclerosis. In the ApoE^–/–^ mice fed with western type diet (WTD), the NINJ1 expression was also upregulated in atherosclerotic aortas, particularly in macrophages. Intriguingly, in the ApoE^–/–^ mice, pharmacological inhibition of NINJ1 reduced the formation of atherosclerotic plaques and lipid accumulation (65). In contrast, as shown independently, genetic deletion of the NINJ1 expression exacerbated the plaque formation in aortas, which may attribute to loss of the anti-atherosclerotic action provided by the soluble form of NINJ1 that is generated upon cleavage by matrix metallopeptidase-9 (MMP-9) from macrophages and suppresses proinflammatory function of macrophages (52).

Studies have also examined the role of pyroptosis in hyperlipidemia-induced atherosclerosis using the LDLR^−/−^ mice. Activation of caspase-1 and caspase-11 and cleavage of GSDMD, as well as elevated infiltration of macrophages and generation of proinflammatory factors, were detected in the atherosclerotic aorta of the LDLR^−/−^ mice fed with a high cholesterol-containing WTD. Importantly, the atherosclerotic plaque formation was assuaged by deleting the GSDMD expression (66). As further revealed in the WTD-fed LDLR^−/−^ mice expressing Janus kinase 2 carrying the proatherogenic V617F mutation (JAK2VF), the AIM2 expression was upregulated in the atherosclerotic plaques, especially in macrophages, and the necrotic core formation was intensified, an effect that was reduced or abrogated by deleting the expression of AIM2, caspase-1, caspase-11 or GSDMD (67). These findings provide further evidence to support the role of hyperlipidemia-induced GSDMD-mediated pyroptosis in atherosclerosis, particularly in macrophage-dependent atherosclerosis progression.

### Pyroptosis of cells induced by ox-LDL

Oxidized LDL (ox-LDL), generated by oxidation of LDL, can accumulate on the arterial walls and induce atherosclerosis (68). A large number of studies have examined ox-LDL-induced pyroptosis of ECs, macrophages and VSMCs and demonstrated the importance of the canonical pyroptosis pathways and, furthermore, multiple molecular mechanisms, by which ox-LDL stimulates such pathways.

#### ECs

As introduced above, loss of ECs represents a common event in the early phase of atherosclerosis. Studies examining ECs from different species and tissues have shown that oxLDL targets ECs and stimulates pyroptosis via the canonical pathways by numerous molecular mechanisms, which for clarity and easiness of discussion are categorized in groups.

**Figure 2:**
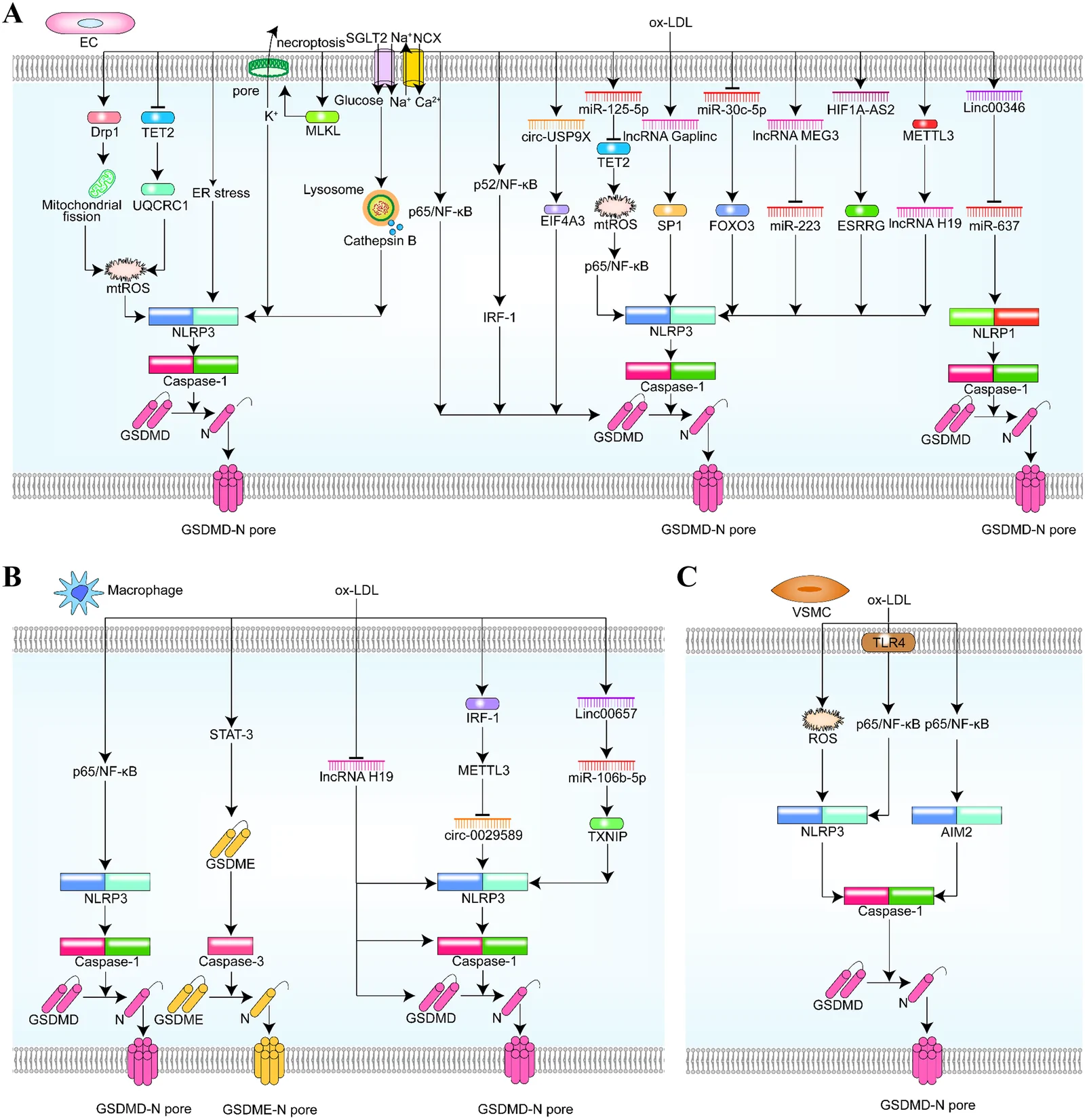
Schematic summary of the molecular and signaling mechanisms for the regulation and activation of pyroptosis pathways by ox-LDL. **(A)** In ECs, ox-LDL can promote mitochondrial ROS generation, via inducing Drp1-mediated mitochondrial fission or downregulation of TET2 methylcytosine dioxygenase-dependent UQCRC expression, to upregulate or activate the NLRP3 inflammasome. Ox-LDL can also induce ER stress, K^+^ efflux through MLKL-mediated necroptosis, or Na^+^ and Ca^2+^ overlading via SGLT2/NCX-mediated to damage lysosome and release lysosomal cathepsin B, to activate the NLRP3 inflammasome. Ox-LDL can upregulate the GSDMD expression via the p65-mediated NF-κB pathway or p52-mediated NF-κB/IRF-1 pathway, and the expression of NLRP3 and NLRP1 via multiple different ncRNA-mediated mechanisms. **(B)** In macrophages, ox-LDL can upregulate the NLRP3 expression via p65-mediated NF-κB pathway to activate caspase-1 and cleave GSDMD, and upregulate GSDME via STAT-3 to activate caspase-3 and cleavage GSDME. Ox-LDL can also upregulate the expression of NLRP3, caspase-1 and GSDMD via different ncRNA-mediated mechanisms, leading to enhanced activation of the NLRP3 inflammasome and caspase-1 and GSDMD cleavage. **(C)** In VSMCs, ox-LDL can induce ROS generation to enhance the expression and/or activation of the NLRP3 inflammasome. Ox-LDL can also upregulate the NLRP3 or AIM2 expression via p65-mediated NF-κB pathway. Drp1, dynamin-related protein 1; TET2, ten-eleven translocation 2; UQCRC1, ubiquinol-Cyt-c reductase core protein 1; mtROS, mitochondrial ROS; ER, endoplasmic reticulum; MLKL, mixed lineage kinase domain-like protein; SGLT2, sodium-glucose cotransporter 2; NCX, Na^+^/Ca^2+^ exchanger; IRF-1, interferon regulatory factor-1; ncRNA, noncoding RNA; SP1, specific protein 1; FOXO3, fork head box O3; ESRRG, estrogen-related receptor gamma; METTL3, methyltransferase-like 3; EIF4A3, eukaryotic translation initiation factor 4A3; TXNIP, thioredoxin-interacting protein; TLR4, toll-like receptor 4.

The first group of molecular mechanisms, by which ox-LDL induces pyroptosis of ECs, is to upregulate and activate the NLRP3 inflammasome (**Figure 2A**). The common route is generation of ROS (69–72). For example, in HUVECs, ox-LDL upregulates the expression of dynamin-related protein 1 (Drp1) (69) and, alternatively, downregulates the expression of ten-eleven translocation 2 (TET2) methylcytosine dioxygenase, which methylates the gene encoding mitochondrial complex III ubiquinol-Cyt-c reductase core protein 1 (UQCRC1) to downregulate the UQCRC1 expression, leading to mitochondrial dysfunction and mitochondrial ROS generation (72). In addition, in HUVECs, ox-LDL can upregulate the expression of ATF6 and GRP78 and induce ER stress to activate the NLRP3 inflammasome (69), or upregulate the expression of mixed lineage kinase domain-like protein (MLKL), the necroptosis executor, and thereby promote necrotic pore formation, resulting in K^+^ efflux for activation of the NLRP3 inflammasome (73). Finally, as also shown in HUVECs, ox-LDL can induce intracellular overloading of Na^+^ and Ca^2+^ via the Na^+^-glucose cotransporter-2 (SGLT2) and Na^+^/Ca^2+^ exchanger (NCX), leading to lysosomal damage and release of cathepsin B into the cytosol to activate the transcriptional NF-κB signaling pathway and thereby upregulate the NLRP3 expression (74).

In the second group of molecular mechanisms, ox-LDL provokes pyroptosis of ECs by upregulating the expression of capase-1 and GSDMD via the NF-κB pathways (**Figure 2A**). As revealed in human aortic endothelial cells (HAECs), ox-LDL can mobilize the p65-mediated NF-κB pathway to upregulate the GSDMD expression (75) and, additionally, activate the p52-mediated NF-κB pathway to upregulate the expression of transcription factor interferon regulatory factor-1 (IRF-1) that drives the expression of caspase-1 and GSDMD (43).

The third or last group of molecular mechanisms, by which ox-LDL stimulates pyroptosis of ECs, is to upregulate the expression and/or activation of NLPR3, NLRP1 and GSDMD via noncoding RNAs (ncRNA), including circular RNA (cirRNA), microRNA (miRNA) and long noncoding RNA (lncRNA) (**Figure 2A**). As shown in HUVECs and HAECs, ox-LDL can stimulate the expression and/or activation of NLRP3 via noncoding RNAs, for example, by enhancing the miR-125a-5p level to inhibit TET2 methylcytosine dioxygenase-mediated abnormal methylation of mitochondrial DNA, leading to mitochondrial ROS generation that upregulates the NLRP3 expression via the p65-mediated NF-κB pathway, as well as activating the NLRP3 inflammasome (75). Ox-LDL can also upregulate the NLRP3 expression by promoting the interactions of lncRNA Gaplinc with transcription factor specific protein 1 (SP1) (76). Furthermore, ox-LDL can modulate the levels of a group of ncRNAs with the so-called “sponge” function to relieve inhibition of the NLRP3 expression (77–79), for example, by reducing the miR-30c-5p level to liberate suppression of the expression of transcriptional regulator FOXO3 that drives the NLRP3 expression (78). Alternatively, ox-LDL can increase the lncRNA MEG3 level to lessen miR-223-mediated inhibition of the NLRP3 expression (79). Similarly, ox-LDL can enhance the NLRP1 expression by increasing the lncRNA Linc00346 level to release miR-637-mediated inhibition of the NLRP1 expression (**Figure 2A**) (77). Finally, ox-LDL can enhance the lncRNA HIF1A-AS2 level to upregulate the expression of estrogen-related receptor gamma (ESRRG) and thereby the expression of NLRP3 (80). As further shown in the HFD-fed ApoE^–/–^ mice, HIF1A-AS2 in extracellular vesicles (EVs) from ox-LDL-treated HUVECs can mediate EVs-induced pyroptosis via upregulating the NLRP3 expression to promote caspase-1 activation and GSDMD cleavage (80). It is noteworthy to mention that ox-LDL can stimulate methyltransferase-like 3 (METTL3) mediated-m6A modification of lncRNA H19 and thereby upregulate the expression of NLRP3, caspase-1 and GSDMD, leading to increased caspase-1 activation, GSDMD cleavage and pyroptosis in HUVECs (59). Ox-LDL can also upregulate the GSDMD expression by stimulating the expression of circ-USP9X and its interactions with eukaryotic translation initiation factor 4A3 (EIF4A3) to stabilize the GSDMD mRNA (81, 82).

In summary, studies have shown a multitude of molecular mechanisms that oxLDL mobilizes to upregulate and/or activate the canonical pyroptosis pathway mediated by NLRP3/NLRP1, caspase-1 and GSDMD to induce pyroptosis of ECs (**Figure 2A**). A recent study has reported that NINJ1 in HUVECs mediates activation of the NF-kB/CXCL-8 signaling pathway in ECs to drive atherosclerosis progression (65).

#### Macrophages

Macrophages play a dual role in the course of many types of inflammatory diseases including atherosclerosis. Macrophages can scavenge harmful substances such as lipoproteins and promote tissue repair and VSMC proliferation to stabilize the plaques, thus exercising an anti-atherogenic role in the early stage (9, 10, 83). However, as the condition progresses to the advanced stage, proinflammatory macrophages in the atherosclerotic plaques secrete matrix-degrading enzymes, elicit death of surrounding cells and promote plaque destabilization and rupture, thus accelerating atherosclerosis progression (9, 10, 83). Increasing evidence indicates that ox-LDL can induce macrophages to undergo pyroptosis by activating the pathways engaging NLRP3, caspase-1, caspase-3, GSDMD or GSDME through multiple molecular mechanisms (**Figure 2B**). For example, ox-LDL can upregulate the expression of NLRP3, ASC and GSDMD in RAW 264.7 cells or the NLRP3 expression via the p65-mediated NF-κB pathway in THP-1-differentiated macrophages (84). In peritoneal macrophages isolated from the ApoE^–/–^ mice, ox-LDL was shown to upregulate the GSDMD expression and activate the NLRP3 inflammasome and caspase-1 (48). Ox-LDL can also upregulate the GSDME expression via signaling molecule transducer and activator of transcription-3 (STAT-3), and promotes capase-3 activation and GSDME cleavage in bone marrow (BM)-derived macrophages (47). Similar to what described above in ECs, ox-LDL can regulate the expression and/or activation of pyroptosis-driving proteins in macrophages via ncRNAs. For example, ox-LDL can upregulate the expression of NLRP3, ASC, caspase-1 and GSDMD by reducing the lncRNA H19 expression in RAW264.7 cells (85). Ox-LDL can also upregulate the NLRP3 expression by inhibiting the circ_0029589 expression via the IRF-1/METTL3-mediated mechanism in human macrophages (86). Moreover, ox-LDL can enhance the linc00657 expression and binding to miR-106b-5p, or the LncRNA MIR17HG expression and binding to miR-301a-3p, thereby alleviating inhibition of the expression of thioredoxin-interacting protein (TXNIP), the activator of the NLRP3 inflammasome, in THP-1-differentiated macrophages (87, 88). As shown in a study discussed above, deletion of the NINJ1 expression in the ApoE^–/–^ mice led to heightened proinflammatory responses of macrophages, thereby aggravating plaque formation, whereas administration of sNINJ1 mimetic peptides can suppress proinflammatory function of macrophages, supporting NINJ1 to play an anti-atherosclerotic role (52).

#### VSMCs

Ox-LDL can also induce pyroptosis of VSMCs through several molecular mechanisms that stimulate the canonical pathways mediated by NLRP3, AIM2, caspase-1 and GSDMD (**Figure 2C**). As shown in mouse VSMCs, ox-LDL can induce ROS generation via undefined mechanisms to upregulate the expression of NLRP3 and ASC to promte caspase-1 activation and GSDMD cleavage (45), and can also enhance the expression of lysosomal cathepsin B to upregulate the NLRP3 expression (55). In rat VSMCs, ox-LDL can upregulate the expression of NLRP3, ASC, caspase-1 and GSDMD via the TLR4/p65-mediated NF-κB pathway (60, 89), whereas, in mouse VSMCs, ox-LDL can induce ROS generation via undefined mechanisms to upregulate the expression of AIM2 and ASC via the p65-mediated NF-κB pathway, inducing caspase-1 activation and GSDMD cleavage in these cells (90).

## Pyroptosis in proatherogenic actions of other risk factors and pathological conditions

**Figure 3:**
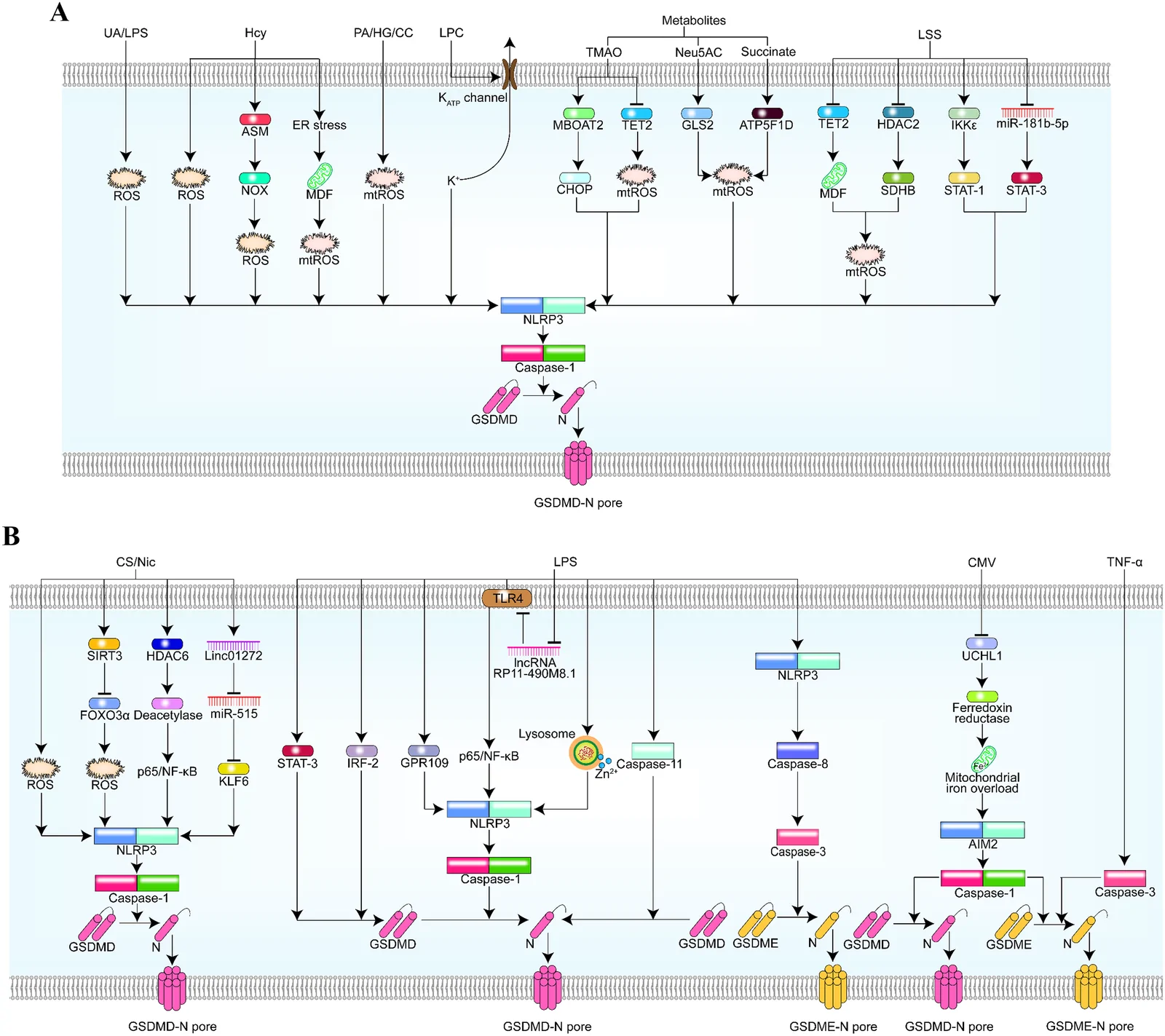
Schematic summary of the molecular and signaling mechanisms for the regulation and activation of the pyroptosis pathways induced by stimuli associated with pro-atherosclerotic risk factors and pathological conditions. **(A)** Endogenous stimuli. UA associated with hyperuricemia in combination with LPS, Hcy associated with hyperhomocysteinemia, PA and HG associated with diabetes, and CC can promote ROS generation, particularly from mitochondria via different mechanisms, to enhance the NLRP3 expression and/or activation. LPC can promote K^+^ efflux through the K_ATP_ channel to activate the NLRP3 inflammasome. Metabolites such as TMAO, Neu5Ac or succinate can induce ER stress or promote mitochondrial ROS generation via TET2, GLS2 or ATP5F1D, to enhance the expression/or activation of NLRP3. LSS can, via inhibiting TET2 or reducing the SDHB expression, promote mitochondrial dysfunction and ROS generation to stimulate the expression/or activation of NLRP3. LSS can also upregulate the NLRP3 expression via IKKe/STAT-1 or STAT-3 pathways. **(B)** Exogenous stimuli. Cigarette smoking extract or nicotine can promote ROS generation via SIRT3/FOXO3a or HDAC6/deacetylase/p65-mediated NF-κB pathway to upregulate the NLRP3 expression. LPS can upregulate the GSDMD expression via STAT-3 or IRF-2, and the NLRP3 expression via GRP109 or p65-mediated NF-κB pathways and activate the NLRP3 inflammasome via inducing lysosomal zinc release. These mechanisms enhance activation of the NLRP3 inflammasome and capase-1 to cleave GSDMD. In addition, LPS can activate caspase-11 to cleave GSDMD. LPS can also increase the NLRP3 expression, which in turn activates caspase-8 and caspase-3 to cleave GSDME. Infection with CMV can inhibit UCHL1 to stimulate ferredoxin reductase and mitochondrial iron ion overload, resulting in activation of the AIM2 inflammasome and caspase-1 to cleave GSDME or GSDMD. TNF-α can upregulate the caspase-3 expression to increase caspase-3-mediated cleavage of GSDME. UA, uric acid; LPS, lipopolysaccharide; Hcy, high homocysteine; ASM, acid sphingomyelinase NOX, NADPH oxidase; MDF, mitochondrial dysfunction; PA, palmitic acid; HG, high glucose; CC, cholesterol crystal; LPC, lysophosphatidylcholine; TMAO trimethylamine N-oxide; MBOAT2, membrane bound glycerophospholipid O-acyltransferase 2; TET2, ten-eleven translocation 2; Neu5Ac, N-acetylneuraminic acid; GLS2, glutaminase 2; ATP5F1D, ATP synthase F1 complex δ subunit; LSS, low shear stress; SDHB, succinate dehydrogenase B; IKK. Inhibitor of B kinase; STAT, signaling molecule transducer and activator of transcription; CS, cigarette smoking extract, Nic, nicotine; SIRT, FOXO3, fork head box O3; HDAC, histone deacetylase; IRF, interferon regulatory factor-1; UCHL1, ubiquitin carboxyl terminal hydrolase 1; CMV, cytomeglovirus; TNF, tumor necrosis factor.

Studies have used the ApoE^−/−^ mouse model to evaluate the role of pyroptosis in mediating the proatherogenic actions of a diversity of other risk factors or pathological conditions (**Table 1**). Studies using cultured cells have gained insights into the pyroptosis pathways and the molecular mechanisms that stimulate such pathways (**Figure 3**).

### Hyperuricemia

Hyperuricemia, due to accumulation of high concentrations of uric acid, is known to exacerbate atherosclerosis (91). In the ApoE^−/−^ mice with hyperuricemia induced by feeding with a high fat and high adenine (HFHA) diet, the expression of NLRP3, caspase-1 and GSDMD was upregulated and caspase-1 was activated. Furthermore, hyperuricemia led to upregulated expression of caspase-1 in ECs and cell death in the atherosclerotic plaques (92). As shown in HUVECs, treatment with high concentrations of uric acid in combination with LPS can promote ROS generation that activates the NLRP3 inflammasome and caspase-1 to induce GSDMD cleavage (92).

### Hyperhomocysteinemia

Hyperhomocysteinemia (HHcy), a condition resulting from accumulation of high concentrations of homocysteine (Hcy), is highly proatherogenic, particularly in the early stage of atherosclerosis, by eliciting pyroptosis of ECs and vascular inflammation (93). In the ApoE^−/−^ mice with HHcy induced by feeding with a high fat and high methionine (HDHM) diet, the expression of NLRP3, caspase-1 and GSDMD was upregulated, and caspase-1 was activated in the atherosclerotic tissues, especially in macrophages (94, 95). As shown in mouse aortic endothelial cells (MAECs), exposure to high Hcy concentrations can induce an intermediate level of ROS via undefined mechanisms to activate the NLRP3 inflammasome and caspase-1 to promote pyroptosis (96). Similarly, in HUVECs, Hcy can upregulate the NLRP3 expression, as well as activating the NLRP3 inflammasome and caspase-1, to promote GSDMD cleavage and pyroptosis (46). Thus, Hcy can induce pyroptosis of ECs via the NLRP3/caspase-1/GSDMD-mediated canonical pathway (**Figure 3A**). Hcy can also promote pyroptosis of macrophages by promoting ROS generation, mainly via two molecular mechanisms (**Figure 3A**). As revealed in THP-1-differentiated macrophages, Hcy can induce lipid raft clustering by enhancing the expression of acid sphingomyelinase (ASM) to activate nicotinamide adenine dinucleotide phosphate (NADPH) oxidases (NOX) and thereby generate ROS, which activates the NLRP3 inflammasome and caspase-1, as well as upregulating the expression of NLRP3, caspase-1 and GSDMD (95). In addition, Hcy can induce ER stress to strengthen the ER-mitochondria communications, leading to mitochondrial Ca^2+^ overloading, mitochondrial dysfunction and mitochondrial ROS generation that activates the NLRP3/caspase-1/GSDMD-mediated canonical pathway (94).

### Diabetes

Diabetes is linked to increased prevalence of atherosclerotic coronary artery diseases (97), particularly type 2 diabetes bears a causative relationship with the vulnerability of advanced atherosclerotic plaques (98). In the HFD-fed WT mice or WTD-fed ApoE^–/–^ mice, streptozotocin (STZ)-induced diabetes led to upregulated expression of NLRP3, ASC and GSDMD in the atherosclerotic aortic sinus or, more specifically, in ECs (99, 100). In the HFD-fed ApoE^–/–^ mice, STZ-induced diabetes also resulted in upregulated expression of NLRP3 and caspase-1 in macrophages (101). Similarly, in diabetic rats with atherosclerosis induced by feeding with a diet containing high levels of fat, cholesterol and carbohydrates, the expression of NRLP3, ASC, caspase-1 and GSDMD was elevated in the aortic tissues (102).

Palmitic acid (PA), rich in high fat diets (103), is known for its ability to cause endothelial dysfunction related to type 2 diabetes by inducing oxidative stress (**Figure 3A**). PA can induce mitochondrial ROS generation to upregulate the expression of NLRP3, ASC, GSDMD and caspase-1 via the TLR4/NF-κB pathway in SVEC4–10 vascular endothelial cells and also in vascular smooth muscle cells (104) and, moreover, activate the NLRP3 inflammasome and caspase-1 (105). In other words, PA can induce pyroptosis via the NLRP3/caspase-1/GSDMD-mediated canonical pathway. As reveled in HUVECs, PA can also induce pyroptosis via the non-canonical pathways, depending upon caspase-4/5-mediated cleavage of GSDMD (106).

Diabetes-related hyperglycemia or high concentrations of glucose predisposes atherosclerosis and can cause pyroptosis of ECs and macrophages by inducing oxidative stress (**Figure 3A**). In HUVECs, exposure to high glucose can induce ROS generation and, in addition, reduce the antioxidant capacity of cells by downregulating the expression of superoxide dismutase (SOD), together leading to an increased ROS level that upregulates the expression of NLRP3 and ASC (107). In human cardiac microvascular endothelial cells, high concentrations of glucose can upregulate the expression of NLRP3 and ASC via the TLR2/MyD88/NF-κB pathway to promote GSDMD cleavage (100). Consistently, the expression of NLRP3, ASC and caspase-1 was upregulated, and pyroptosis was induced in macrophages isolated from diabetic rats with atherosclerosis (102). In rat BM-derived and THP-1-differentiated macrophages, high concentrations of glucose in combination with ox-LDL can stimulate the expression of NLRP3, ASC and caspase-1 (101, 102). Mechanistically, treatment with glucose and ox-LDL can increase the expression of LncRNA-metastasis associated lung adenocarcinoma transcript 1 (MALAT1), which is strongly related to diabetes complications, leading to increased NLRP3 expression (102). Pyroptosis induced by glucose and ox-LDL in combination was suppressed by deleting the MALAT1 expression (102). Thus, diabetes-related high level of glucose on its own or in combination with ox-LDL can cause pyroptosis of ECs and macrophages by inducing oxidative stress to stimulate the NLRP3/caspase-1/GSDMD-mediated canonical pathway.

### Metabolites

Several metabolites, exemplified by trimethylamine N-oxide (TMAO), N-acetylneuraminic acid (Neu5Ac) and succinate, have been strongly implicated in mediating atherosclerosis. TMAO, a metabolite produced by intestinal flora, has been recognized as an independent risk factor for atherosclerosis and related conditions. In the ApoE^−/−^ mice, feeding with a TMAO-containing diet led to upregulated NLRP3 expression in ECs, elevated lipid deposition and accelerated atherosclerotic plaque formation in the aortic roots (108). Neu5Ac is a metabolite produced by the hexosamine-sialic acid pathway branching from glucose metabolism and, treatment of the ApoE^−/−^ mice with Neu5Ac aggravated atherogenic changes, manifested by enlarged plaque lesion, enhanced infiltration of macrophages and upregulated NLRP3 expression in the atherosclerotic aortic roots, particularly in CD31-positive ECs (109). Succinate, an essential circulating metabolite within the tricarboxylic acid cycle, is known as a proinflammatory molecule. Both in the serum of patients with coronary heart disease and in the serum of the HFD-fed ApoE^−/−^ mice, the succinate level is positively correlated with the levels of IL-6 and IL-18 (51). Thus, these findings point to TMAO, Neu5Ac and succinate for their proatherogenic role.

As shown in HUVECs, TMAO can upregulate the NLPR3 expression by activating the PERK/eIF2α/CHOP signaling pathway through the membrane bound glycerophospholipid O-acyltransferase 2 (MBOAT2), leading to pyroptosis via the NLRP3/caspase-1/GSDMD-mediated canonical pathway (108). TMAO can also inhibit the TET2 expression to methylate the gene encoding cytochrome b, a component of mitochondrial complex II, resulting in increased mitochondrial ROS generation to upregulate the expression of NLRP3, caspase-1 and GSDMD, thereby promoting caspase-1 activation, GSDMD cleavage and pyroptosis in vascular ECs (110). As shown in HUVECs and also EA.hy926 cells, Neu5Ac can activate the SIRT3/FOXO3a/c-Myc signaling pathway to upregulate the expression of glutaminase 2 (GLS2) and thereby enhance generation of α-ketoglutarate (α-KG), which in turn promotes mitochondrial dysfunction and mitochondrial ROS generation to activate the NLRP3/caspase-1/GSDMD-mediated canonical pathway (109). Accumulation of succinate in HUVECs induced by treatment with diethyl butyl malonate was shown to downregulate the expression of ATP synthase F1 complex δ subunit (ATP5F1D) and, as a result, promote mitochondrial dysfunction and mitochondrial ROS generation that enhances the expressions and/or activation of the NLRP3 inflammasome to induce caspase-1 activation, GSDMD cleavage and pyroptosis (51). These findings support induction of pyroptosis of ECs via the NLRP3/caspase-1/GSDMD-mediated canonical pathways by multiple molecular mechanisms plays an important role in the proatherogenic actions of TMAO, Neu5Ac and succinate (**Figure 3A**).

### Low shear stress

Low shear stress, commonly occurring at the bifurcations of curved blood vessels, is another proatherogenic factor. In the ApoE^–/–^ mice fed by a high cholesterol diet (HCD), activation of the inhibitor of κB kinase (IKK) ϵ, an important regulator of immune response, can increase lipid deposition and upregulate the expression of intimal NLRP3 in the atherosclerotic plaques at the curved vessel bifurcations, which were abolished by EC-specific deletion of the IKKϵ expression (111). Consistently, as shown in HUVECs, exposure to low shear stress can activate IKKϵ, upregulate the NLRP3 expression, and induce caspase-1 activation, GSDMD cleavage and pyroptosis (111). Furthermore, low shear stress has been shown to upregulate the expression of NLRP3 and GSDMD via three molecular mechanisms (**Figure 3A**). Firstly, low shear stress can downregulate the expression of TET2 methylcytosine dioxygenase or, alternatively, recruit histone deacetylase 2 (HDAC2) to enhance the expression of succinate dehydrogenase B (SDHB), a component of mitochondrial complex II, thereby promoting mitochondrial ROS generation to upregulate the expression of NLRP3 and GSDMD (112). Secondly, low shear stress can activate IKKϵ and STAT-1 to drive the NLRP3 expression (111). Lastly, low shear stress can disrupt the binding of miR-181b-5p to STAT-3 to enhance the STAT-3 expression and thereby upregulate the NLRP3 expression (113). Therefore, low shear stress can elicit pyroptosis of ECs via stimulating the pathway depending upon NLRP3 and GSDMD. It is assumed that NLRP3 activates caspase-1 to cleave GSDMD, but the supporting evidence still awaits.

### Cigarette smoking

Cigarette smoking is a notorious perpetrator of atherosclerosis and related CVDs (114, 115). Nicotine is the principal toxic component in extracts of cigarette smoking. In the HFD-fed ApoE^−/−^ mice, oral administration of nicotine (100 μg/mL; LD50 ~ 50 mg/kg body weight) was shown to promote cell death, as well as upregulating the expression of NLRP3 and ASC and inducing caspase-1 activation and GSDMD cleavage in atherosclerotic aortic tissues and, furthermore, accelerate the atherosclerotic plaque formation (116–118). More specifically, exposure to nicotine can induce cell death and upregulate the expression of NLRP3, ASC and caspase-1 in ECs (117), or enhance the expression and activation of caspase-1 in macrophages in the atherosclerotic aortic tissues (82, 116). Consistently, as shown in HUVECs, treatment with extracts of cigarette smoking or nicotine can induce pyroptosis of ECs. Mechanistically, extracts of cigarette smoking can stimulate ROS generation and thereby upregulate the expression of NLRP3 and ASC and/or activate the NLPR3 inflammasome and capase-1 to induce GSDMD cleavage and pyroptosis. Such pyroptosis was attenuated by treatment with the anti-oxidant N-acetyl-l-cysteine (NAC) or the NLPR3 inflammasome inhibitor MCC950 (119). Similarly, in HAECs, exposure to nicotine can induce ROS generation, leading to upregulated expression of NLRP3 and ASC, activation of the NLRP3 inflammasome and pyroptosis. Likewise, nicotine-induced pyroptosis was suppressed by treatment with NAC or MCC950, and also by siRNA-mediated knockdown of the expression of NLRP3 or ASC (117). Thus, cigarette smoking can cause pyroptosis of ECs by inducing ROS generation to stimulate the NLPR3/caspase-1/GSDMD-mediated pathway (**Figure 3B**).

Nicotine can also induce pyroptosis of macrophages via the NLRP3/caspase-1/GSDMD-mediated canonical pathway by activating a multitude of molecular mechanisms (**Figure 3B**). Firstly, as shown in BM-derived macrophages, exposure to nicotine can induce ROS generation to activate the NLRP3 inflammasome and caspase-1, and promote GSDMD cleavage and pyroptosis, in addition to enhancing lipid phagocytosis to facilitate foam cell formation (118, 120). Secondly, nicotine can upregulate the sirtuin 3 expression to downregulate the FOXO3α expression, resulting in enhanced ROS generation to upregulate the expression of NLRP3 and ASC and activate the NLRP3 inflammasome in THP-1-differentiated macrophages (121). Thirdly, nicotine can upregulate the expression of HDAC6 that increases deacetylation of p65, leading to activation of the p65-mediated NF-κB pathway to drive the NLRP3 expression in RAW264.7 cells (116). Lastly, as revealed in THP-1-derived macrophages, nicotine can stimulate the linc01272 expression and its binding with miR-515 to relieve suppression of the KLF6 expression and thereby upregulate the NLRP3 expression, giving rise to increased caspase-1 activation and GSDMD cleavage (44).

In summary, cigarette smoking can induce pyroptosis of ECs and macrophages by marshalling multiple molecular mechanisms that upregulate and/or activate the NLRP3/caspase-1/GSDMD-mediated canonical pathway (**Figure 3B**).

### Infection

Infection is a common pathological condition that increases the susceptibility to atherosclerosis and induces pyroptosis, as a result of proinflammatory reactions (122). For example, exposure to LPS from bacterial infection can promote atherosclerosis in mice and rats (123), and mouse cytomeglovirus (CMV) can cause atherosclerosis-related damage and, moreover, upregulate the expression of AIM2, GSDMD and GSDME in the endothelium and mesothelium in mice (124). In the HFD-fed ApoE^–/–^ mice, injection of LPS at a low dose (1 ng/mL) was shown to promote infiltration of macrophages, upregulate the expression of caspase-1, caspase-11 and GSDMD in the aortic roots or the GSDMD expression in ECs, induce GSDMD cleavage and aggravate vascular endothelial inflammation and atherosclerotic plaque formation. Importantly, LPS-induced vascular endothelial inflammation and atherogenesis were alleviated by deleting EC-specific expression of GSDMD (70).

Studies examining cultured ECs provide evidence that LPS can induce pyroptosis of ECs via the NLPR3/caspase-1/GSDMD-mediated canonical pathway and shed light on the underlying molecular mechanisms (**Figure 3B**). For example, in HUVECs, LPS can upregulate the GSDMD expression by activating STAT-3 (70). LPS can also reduce the lncRNA RP11-490M8.1 level to relax its inhibition on the expression of TLR4 and p65, thereby enhancing the TLR4/p65-mediated NF-κB pathway to drive the expression of NLRP3, ASC and caspase-1 (125). In HAECs, LPS can upregulate the expression of NLRP3 and ASC and thereby activate the NLRP3 inflammasome and caspase-1 (126).

LPS can elicit pyroptosis of macrophages through multiple molecular mechanisms that activate or enhance the NLRP3/caspase-1/GSDMD-mediated canonical pathway or the caspase-11/GSDMD-mediated non-canonical pathway (**Figure 3B**). LPS can upregulate the expression of NLRP3 and caspase-1 in BM-derived macrophages (127) and, more specifically, in RAW264.7 cells, upregulate the NLRP3 expression via the TLR4/p65-mediated NF-κB pathway (128) or the expression of NLRP3 and GSDMD via the G protein-coupled receptor 109A (GPR109A) (129). LPS can also upregulate the NLRP3 expression via the TLR4/p65-mediated NF-κB pathway (128) or the Nrf2/HO-1 pathway in THP-1 cells (130), and enhance IRF-2-mediated expression of GSDMD in immortalized macrophages (131). LPS-induced pyroptosis in THP-1 cells was alleviated by treatment with disulfiram, which covalently modifies Cys^192^ in mouse GSDMD or Cys^191^ in human GSDMD, respectively, to prevent GSDMD cleavage (132). In addition, LPS can activate caspase-11 to cleave GSDMD, particularly facilitate GSDMD-N oligomerization by promoting ROS generation and thereby oxidizing Cys^192^ in mouse GSDMD in immortalized macrophages (133). Moreover, LPS in combination with nigericin was shown to induce pyroptosis via promoting release of lysosomal Zn^2+^ through the TRPML1 channel that activates the NLRP3/caspase-1/GSDMD-mediated canonical pathway to execute pyroptosis in J774A.1 cells (50). It is worth mentioning the findings that LPS can provoke pyroptosis in GSDMD-deficient macrophages, by engaging NLRP3, caspase-3 and GSDME (134). In addition, in BM-derived macrophages from the GSDMD-KO mice expressing the constitutive NLRP3 D301N mutant inflammasome, LPS promoted caspase-3 activation, GSDME cleavage and pyroptosis, and furthermore, such LPS-induced pyroptosis was reduced by deleting the GSDME expression (134). These results led to the proposal that when GSDMD is absent or inhibited, LPS can trigger pyroptosis of macrophages via the NLRP3/caspase-3/GSDME-mediated pathway that normally remains silent or redundant.

There is evidence that CMV infection can induce pyroptosis of ECs (135). As shown in HUVECs and HAECs (124), human CMV can downregulate the expression of ubiquitin carboxyl terminal hydrolase 1 (UCHL1) to reduce degradation of ferredoxin reductase (FDXR), a mitochondrial protein important in regulating mitochondrial iron ion homeostasis. As a result, iron ions are accumulated in mitochondria, leading to increase in the expression of AIM2, activation of AIM2 and capase-1, and cleavage of GSDMD and GSDME (124) (**Figure 3B**).

### Proinflammatory lipids and factors

Cholesterol crystals are a potent inducer of inflammation and present in the advanced atherosclerotic plaques. Cholesterol crystals have been proposed to play a proatherogenic role via inducing ROS generation, lysosomal damage and mitochondrial dysfunction to promote activation of the NLRP3 inflammasome and pyroptosis (136). However, cholesterol crystals-elicited pyroptosis pathways in atherosclerosis are not fully defined.

Lysophosphatidylcholine (LPC), a phospholipid abundant in human blood, possesses a proinflammatory activity. As shown in THP-1-differentiated macrophages, LPC can promote K^+^ efflux through the ATP-sensitive K^+^ channel (K_ATP_) to activate the NLRP3 inflammasome and thereby the NLRP3/caspase-1/GSDMD-mediated canonical pathway to induce pyroptosis (137) (**Figure 3A**).

Proinflammatory factors such as TNF-α are suggested to accelerate atherosclerosis progression. As shown in HUVECs, TNF-α can induce pyroptosis of ECs by activating the NLRP3 inflammasome and caspase-1 to cleave GSDMD and, in addition, caspase-3 to cleave GSDME (138) (**Figure 3B**).

To summarize, studies provide a body of evidence to support the role of pyroptosis of ECs, macrophages and VSMCs via multiple gasdermin-mediated pathways in atherosclerotic settings, with the NLRP3/caspase-1/GSDMD-mediated canonical pathway being the most common, if not dominant, pathway. Moreover, *in vitro* studies have identified a large number of distinctive molecular mechanisms that activate or regulate such pyroptosis pathways. However, it is important to emphasize that the relevance or contribution of many of these pathways and mechanisms in the pathogenesis and progression of atherosclerosis need further investigations.

## Pyroptosis in atherosclerosis: therapeutic opportunities

While researches continue to gain more mechanistic insights into the proatherogenic role of pyroptosis, there is a growing interest in the therapeutic potential of targeting pyroptosis-driving pathways and mechanisms to intervene atherosclerosis and related CVDs.

### Repurposing of hypoglycemic or cholesterol-reducing medicines

There is an interest in repurposing metformin, the classical hypoglycemic drug, and atorvastatin, a medicine used to reduce blood cholesterol, for the treatment of atherosclerosis. In the HFD-fed ApoE^−/−^ mice with STZ-induced diabetes, oral feeding of metformin in a dosage of 300 mg/kg/day for 20 weeks can alleviate NLRP3-mediated pyroptosis and atherosclerotic plaque formation (139), and treatment with atorvastatin via gavage in a dosage of 3 mg/kg/day for 6 weeks can suppress lipid deposition and the expression of GSDMD, NLRP3 and caspase-1 and atherosclerotic plaque formation (140). As also shown in the HFD-fed ApoE^−/−^ mice, administration via oral gavage of Tangzhi Qing, a traditional Chinese herbal medicine prescribed to address glucose and lipid metabolic abnormalities, in a dosage of 3 mg/kg/day for 6 weeks can downregulate the expression of ASC, caspase-1 and GSDMD and alleviate atherosclerotic plaque formation (141).

### Immunomodulatory interventions targeting proinflammatory cytokines

The immunomodulatory strategy, as an alternative to the conventional lipid-lowering treatment, has gained considerable attention in managing atherosclerosis and related conditions such as chronic coronary disease for the inflammatory nature of these conditions. For example, in the Canakinumab Anti-inflammatory Thrombosis Outcome Study (CANTOS), treatment of post-MI patients with canakinumab, a human anti-IL-1β monoclonal antibody, given subcutaneously in a dosage of 150 mg once every 3 months can reduce the levels of IL-1β, IL-6 and high sensitivity C-reactive protein (142). Oral intake of colchicine, an anti-inflammatory drug, in a dosage of 0.5 mg/day for 2 or 5 years, can mitigate the occurrence of major adverse cardiac or cerebrovascular events in chronic coronary disease patients (143, 144). It is worth pointing out that not all of the immunomodulatory interventions generate desired benefits, for example, anakinra, a recombinant antagonist of the IL-1 receptor, being ineffective in reversing the process of atherosclerotic plaque formation in acute coronary syndromes (145). In the Cardiovascular Inflammation Reduction Trial (CIRT), administration of methotrexate in patients with stable atherosclerosis reduced neither the levels of IL-1β, IL-6 and high sensitivity C-reactive protein nor the cardiovascular events including cardiovascular death, nonfatal myocardial infarction, nonfatal ischemic stroke and coronary revascularization (146).

### Inhibitors targeting the NLRP3/capase-1/GSDMD-mediated pyrotosis pathway

As noted from the discussed above, increasing evidence supports an important role for the NLRP3/capase-1/GSDMD-mediated pathway in pyroptosis of ECs, macrophages and VSMCs in the atherosclerotic settings. Therefore, several recent studies have assessed the effectiveness of inhibitors targeting key proteins in this canonical pyroptosis pathway, including NLRP3, caspase-1 and GSDMD, in alleviating atherosclerosis using rodent models. For example, in the WTD-fed ApoE^−/−^ mice, intraperitoneal administration of MCC950 in a dosage of 10 mg/kg/day for 12 weeks reduced GSDMD cleavage, protected loss of VSMCs and macrophages and, importantly, alleviated the atherosclerotic plaque formation (147, 148). MCC950 is thought to have high target specificity and low off-target toxicity, but long-term systemic administration may impair NLRP3-mediated innate immunity (149, 150). In the Colchicine Cardiovascular Outcomes Trial (COLCOT), the beneficial effects of oral intake of colchicine in a dosage of 0.5 mg/day for 2 years in patients experiencing MI have been attributed to inhibition of the NLRP3 inflammasome (151). Colchicine can also inhibit leukocyte chemotaxis and phagocytosis (152), risking the systemic anti-infection capacity. In the HFD-fed ApoE^–/–^ (45) and LDLR^–/–^ mice (56), intragastric gavage of the caspase-1 inhibitor VX-765 in a dosage of 50 mg/kg/day for 12 weeks attenuated the formation of necrotic cores and atherosclerotic plaques. The intensive immunosuppression of VX-765 can also potentially compromises the ability of clearing bacteria and viruses (153, 154). In the HFD-fed ApoE^–/–^ mice, oral feeding of disulfiram, in a dosage of 100 mg/kg/day for 15 weeks to inhibit GSDMD cleavage, mitigated the formation of necrotic cores and atherosclerotic plaques (155). Also shown in the HFD-fed ApoE^–/–^ mice, treatment of necrosulfonamide, by intraperitoneal injection with single dosage of 20 mg/kg or by oral gavage with 5 mg/kg/day for 30 days to prevent GSDMD-N from forming the pyroptotic pore (156) or caspase-1 activation (157), suppressed the formation of atherosclerotic plaques and infiltration of macrophages into the plaques (158). Systemic administration of such GSDMD inhibitors however has a narrow therapeutic window and high cytotoxicity (147, 159). In summary, these studies provide further evidence to support the importance of the NLRP3/caspase-1/GSDMD-mediated pyroptosis pathway in the pathogenesis and progression of atherosclerosis and demonstrate the therapeutic potential of targeting such a canonical pyroptosis pathway to treat atherosclerosis. However, further efforts are required to develop therapeutics targeting such pathways to effectively treat atherosclerosis while minimizing the risk of severe infections due to chronic systemic immunosuppression.

### Antioxidants

It is also noted from the discussion above that ROS generation serves as a critical intermediate mechanism mediating pyroptosis of ECs, macrophages and VSMCs induced by a diversity of proatherogenic risk factors and pathological conditions. Recent studies have evaluated the effectiveness of agents with an antioxidant capacity in inhibiting atherosclerosis-related pyroptosis of ECs and macrophages *in vitro* and *in vivo*. For example, in HUVECs, treatment with 10 mM NAC for 24 hours reduced uric acid/LPS-induced pyroptosis (92) and treatment with natural extract flavonoid dihydromyricetin at 40 μM for 24 hours attenuated PA-induced pyroptosis (105). Intraperitoneal injection of melatonin in a dosage of 10 mg/kg/day for 2 weeks mitigated cigarette smoking extract-induced pyroptosis of ECs in rats (119). Oral gavage with isoorientin in a dosage of 20 mg/kg/day for 12 weeks lessened pyroptosis of macrophages in the HFD-fed ApoE^−/−^ mice (63). It is important to emphasize how effective these antioxidant agents improve atherosclerosis in patients remains an open question. Of notice, a recent randomized, double-blinded, placebo-controlled clinical trial, testing the effectiveness of selenite or selenite-enriched yeast in a cohort of patients with atherosclerosis, has reported that selenite supplementation with 200 μg/day for 8 weeks can improve the antioxidant defense and downregulate the expression of NLRP3 and multiple other proinflammatory and pyroptosis-related proteins in atherosclerotic patients (160).

## Conclusions and perspectives

Atherosclerosis and related CVDs represent highly unmet clinical challenges. As discussed above, analysis of tissues from patients with atherosclerosis and related cardiovascular conditions and also from rodent disease models provide consistent evidence to support a significant role of pyroptosis in the pathogenesis and progression of atherosclerosis, and in the proatherogenic actions of diverse risk factors and pathological conditions. Moreover, mechanistic studies have revealed a multitude of molecular mechanisms that drive atherosclerosis-related pyroptosis of ECs, macrophages and VSMCs. In short, recent years have witnessed substantial advances in understanding the role of pyroptosis in atherosclerosis and gaining substantial insights into the pyroptosis pathways, particularly the important role of the NLRP3/caspase-1/GSDMD-mediated pathway, and the molecular mechanisms activating such pathways. It remains less understood with respect to the roles of other canonical pathways and non-canonical pathways. As pointed out above, investigations are required to better understand the contribution of pyroptosis of different cell types via different pathways activated or regulated by different molecular mechanisms in different stages of atherosclerosis. PCD via modalities other than pyroptosis occur in the atherosclerotic settings, and emerging evidence indicates communications of pyroptosis with necroptosis (73) (**Figure 2A**), autophagy (58), ferroptosis (161, 162), but currently it remains poorly understood whether such cross-talk are synergic or redundant in atherosclerosis. A better understanding of the pathways and molecular mechanisms driving pyroptosis and other cell death in atherosclerosis pathogenesis and progression is vital for developing effective therapeutics to treat atherosclerosis and related CVDs.
